# Towards a Multidimensional Understanding of “Being Relative” in Residential Long-Term Care: Findings From a Qualitative Multiperspective Study

**DOI:** 10.1177/23333936261454128

**Published:** 2026-06-23

**Authors:** Rouven Brenner, Heidrun Gattinger, Hanna Mayer

**Affiliations:** 1 University of Vienna, Austria; 2 Eastern Switzerland University of Applied Sciences, St. Gallen, Switzerland; 3 Karl Landsteiner University of Health Sciences, Krems, Austria

**Keywords:** relatives, residential long-term care, person-centred care, institutional presence, qualitative research, Switzerland

## Abstract

Relatives are constitutive to residential long-term care, yet are often conceptualised through a functional lens that focuses on tasks or burden. This qualitative multiperspective study explores the ontological condition of “being relative” beyond these functional categories. Analysing 30 “Integrated Encounter Analyses” from two Swiss facilities, which synthesised participant observations and interviews, we identified eight constitutive dimensions organised into Situational, Relational, and Spatial-Temporal meta-dimensions. Findings reveal “being relative” not as a static role but as a dynamic movement across continua – oscillating between alienation and resonance, and institutional subjection and strategic agency. We conclude that “being relative” is a distinct existential condition characterised by permanent liminality – dwelling in the in-between rather than transitioning through it. The institutional context fundamentally shapes this existential condition: agency manifests not as resistance against but as quiet negotiation within constraints. Institutions must therefore validate the existence of this threshold and support relatives’ navigational competencies rather than forcing binary categorisations of visitors or partners.

## Background

Relatives shape the everyday lives of people living in long-term care facilities and, together with residents and nursing staff, form an interdependent triad (Bauer, 2006; Bauer & Nay, 2003; Koster & Nies, 2022). Although the nature and frequency of relatives’ presence vary, their ongoing connection to residents constitutes a fundamental dimension of institutional care (Barken & Lowndes, 2018; Gaugler, 2005; Tornatore & Grant, 2004). Understanding how relatives experience their presence in these settings is therefore essential for person-centred approaches that recognise all persons involved in the care triad. “Being relative” – not “being *a* relative” – is not an external addition to institutional life, but a constitutive element of it. The deliberate omission of the article signals the study’s central concern: not the social role that kinship confers, but the ontological condition of dwelling within and toward the institutional world.

Despite this importance, relatives are often conceptualised through a functional lens, emphasising tasks, roles, and participation patterns (Chang & Yu, 2013; Hovenga et al., 2022; Saga et al., 2021; Westin et al., 2009). Research has predominantly focused on what relatives do – visiting, providing information, assisting with care – and on how they feel about these activities, particularly regarding burden, stress, or satisfaction (Birtha & Holm, 2017; Carlsen & Lundberg, 2018; Puurveen et al., 2018). While recent studies increasingly recognise relatives as partners in care, this partnership is typically defined by contributions to care outcomes rather than by the relatives’ own lived experiences as persons (Bjørge et al., 2023; Holmberg et al., 2020; Hovenga et al., 2022). Even studies that have explored the meaning of transitions (Kellett, 1999) and identity changes (Puurveen et al., 2018) illuminate the function of relatives rather than the ontology of “being relative.” This leaves a fundamental ontological question: not what relatives do or how burdened they are, but how they exist within institutional contexts (Darbyshire & Oerther, 2021; Mackey, 2005). How is the condition of “being relative” fundamentally constituted in institutional settings? Through what dimensions of being does this existential condition manifest in everyday life? This ontological gap limits both theoretical development and practical approaches to person-centred care.

McCormack and McCance’s (2021) conceptualisation of personhood provides the ontological foundation for this study. They define personhood not as a fixed attribute but as a dynamic state constituted through five interrelated modes of being: being with self, being in relation, being in a social world, being in place, and being in time. While predominantly applied to residents and patients, this framework can equally illuminate relatives’ experiences in institutional contexts.

To fully understand “being relative,” however, these abstract modes of being must be grounded in the concrete reality of institutional encounters. Philosophically, personhood is not a pre-existing essence but emerges through genuine encounter with others (Buber, 1923), suggesting that “being relative” is fundamentally constituted in everyday encounters with residents, staff, and the environment. At the same time, institutional contexts fundamentally structure the possibilities and constraints of these encounters (Goffman, 1961). Everyday encounters thus serve not merely as a unit of care but as the empirical ground on which the existential condition of “being relative” manifests – in the tension between functional roles and human connection, between institutional constraints and personal agency. Understanding how these tensions unfold across situational, relational, and spatial-temporal dimensions requires both adequate methods and theoretical frameworks capable of engaging with the phenomenon’s multidimensional complexity.

Current understanding of relatives’ experiences relies predominantly on retrospective interviews and surveys (Chang & Yu, 2013; Gaugler, 2005; Holmberg et al., 2020; Puurveen et al., 2018; Westin et al., 2009). While valuable, these methods provide limited access to the subtle, situated, and embodied practices through which “being relative” is enacted in real-time (Balcom et al., 2021). To understand “being relative” as an ontological condition rather than a functional role, research must capture the relational, spatial, and temporal dimensions as they unfold in actual encounters. This necessitates multiperspective observational approaches that make visible what often remains implicit in retrospective accounts: the situated interactions and contextual conditions through which “being relative” is constituted.

Drawing on the Person-Centred Nursing Framework’s (McCormack & McCance, 2021) conception of personhood as constituted through five “modes of being,” this study specifically focuses on the three dimensions that manifest most visibly in interpersonal encounters: being in relation (interpersonal connections and relational patterns), being in place (negotiating spatial dimensions and positioning), and being in time (experiencing and enacting temporal rhythms). These three dimensions were selected because they are directly observable within everyday encounters between relatives and nursing staff. In contrast, the other dimensions (being with self, being in a social world) remain less accessible to purely observational inquiry.

The aim of this study is therefore to understand the constitutive conditions of “being relative” in institutional long-term care by exploring how this condition manifests across the relational, spatial, and temporal dimensions of everyday encounters. Specifically, the study addresses three questions:

1. The relational dimension: How do relatives experience interpersonal connections?
2. The spatial dimension: How is space negotiated and inhabited within the institution?
3. The temporal dimension: How are temporal rhythms and institutional time experienced?

## Methods

We followed an interpretive multiperspective qualitative approach to explore “being relative” in everyday encounters between relatives and nursing staff. While the care triad – resident, relative, and nursing staff – constitutes the relational reality of residential long-term care, this study deliberately focuses on dyadic encounters between relatives and nursing staff within this triadic context. The resident constitutes both the occasion and the legitimation for these dyadic encounters – it is the shared concern for the resident that brings relatives and nursing staff into contact. This design decision reflects both the study’s ontological interest in how “being relative” is enacted in direct interpersonal encounters and its positioning within the larger BE-REAL (BEing-RElative: A person-centred perspective on relative in Long-term care) research programme, which investigates these relational dynamics across multiple studies. Drawing on McCormack and McCance’s (2021) Person-centred Framework, we focused on three dimensions that are particularly manifest in interpersonal encounters: being in relation, being in place, and being in time. We integrated three data collection methods – participant observation (Spradley, 2013), follow-up conversations with relatives, and walking interviews with nursing staff (Evans & Jones, 2011; Kusenbach, 2018) – to capture both the observable dynamics of encounters and participants’ situated reflections on these experiences. The term “multiperspective” thus refers to the triangulation of these methodologically distinct vantage points on the same encounter, rather than to the inclusion of all members of the care triad as interview participants.

We employed Reflexive Thematic Analysis (Braun & Clarke, 2019, 2021; Byrne, 2022), selected for its capacity to generate interpretive insights grounded in experiential data while acknowledging the active, reflexive role of researchers in constructing meaning. The analytical process followed an abductive approach: dimensional coding applied predetermined dimensions from the Person-Centred Framework, while Reflexive Thematic Analysis allowed themes to be constructed inductively from the data. Our analytical approach prioritised progressive abstraction: through the triangulation of multiple data sources and systematic analytical steps, we elevated the findings to a conceptual level, identifying the constitutive dimensions of “being relative” rather than describing individual participants’ perspectives.

### Setting

We conducted the study in two residential long-term care facilities in Switzerland between January and June 2025. Both facilities were similar in size, each providing approximately 105 beds for older adults requiring 24-hour nursing care. In consultation with facility leadership, we selected one care unit in each facility as the primary field site, identifying units where staff and residents could accommodate the research presence and where relatives’ visits were frequent enough to enable sustained observation.

We purposefully selected these two settings to explore contrasting relational contexts. Facility A, located in a rural, mountainous region, served a local population in which nursing staff often knew relatives prior to admission and used informal address forms. Facility B, in a more urbanised region, served a more mobile population where pre-existing relationships were less common and formal address forms predominated. This contrast enabled examination of whether pre-existing relationships influenced relational dynamics.

### Participants

We used purposive sampling to recruit relatives and nursing staff, prioritising thematic depth and richness over demographic variation (Braun & Clarke, 2021). The sampling design followed an intensive case logic: rather than recruiting a large number of relatives observed once, we selected a smaller number of relatives and observed each across multiple encounters with different nursing staff members. This design generated analytical depth by systematically varying dyadic constellations within each case. The higher number of nursing staff relative to relatives reflects this logic: each relative was observed in encounters with several different staff members, enabling analysis of how relational dynamics varied across caregiving dyads.

We selected relatives based on frequency of visits and willingness to participate in multiple data collection sessions. Prior to recruitment, we distributed written study information to all relatives and nursing staff and conducted information sessions at team meetings. During a 2-week recruitment phase at each facility, we identified potential participants through consultations with primary nurses, who either introduced the researcher directly during visits or facilitated initial contact by informing relatives about the study.

We recruited 5 relatives and 10 nursing staff members. All participants engaged in multiple data collection sessions at varying frequencies, enabling repeated observations of encounters and temporal variation in experiences. Each relative participated in 4 to 11 observation sessions, while each nursing staff member was observed across 1 to 7 encounters, yielding 30 observed encounters in total, each comprising an observation, a follow-up conversation, and a walking interview. Five relatives declined initial participation (three citing age-related reasons, two citing lack of time), and one relative withdrew during the study due to the death of their mother. No nursing staff declined participation or withdrew.

Participating relatives (*n* = 5) included adult children (*n* = 3) and spouses (*n* = 2), aged 59–81 years. Their family members had been living in the facility for 18 months to 2 years, and relatives visited daily to several times per week. Nursing staff (*n* = 10; 9 women, one man) aged 37–63 years represented different professional roles (registered nurses, licensed practical nurses, care assistants, including staff with leadership responsibilities) and had long-term care experience ranging from 1.5 to 30 years. Table 1 provides an aggregated overview of participant characteristics. Given the small sample size and the situationally detailed encounter descriptions in the findings, we present characteristics in aggregated form to protect participant anonymity and prevent re-identification.

**Table 1.** Participants’ Characteristics.

Characteristic	Relatives (*n* = 5)	Nursing staff (*n* = 10)
Gender	Not reported for anonymity	9 women, 1 man
Age range	59–81 years	37–63 years
Relationships to resident / Professional role	Adult children (*n* = 3) Spouses (*n* = 2)	Registered nurses (incl. leadership roles), licensed practical nurses, care assistants
Duration of the resident’s stay /LTC experience	18 months–2 years	1.5–30 years
Visit frequency /Employment	Daily to several times per week	80%–100%
Observation sessions per Person	4–11	1–7

*Note.* Characteristics are presented in aggregated form to protect participant anonymity given the small sample size and situationally detailed encounter descriptions in the findings. LTC = long-term care. “Observation sessions per person” refers to the number of Integrated Encounter Analyses in which each participant was involved. The unequal ratio of relatives to nursing staff reflects the study’s intensive case design: each relative was observed across encounters with varying staff members to capture relational variation within dyadic constellations.

We recruited all relatives who visited frequently, were willing to participate multiple times, and provided consent. Recruitment proceeded alongside data collection, with assessment of thematic sufficiency (Braun & Clarke, 2021) guiding the decision to conclude recruitment. Consistent with Reflexive Thematic Analysis, thematic sufficiency was not assessed through counting recurring codes but through evaluating whether the data and our analytical engagement with them enabled a rich, nuanced account of the constitutive dimensions of “being relative.”

### Researcher Positioning

The first author [RB] – a male doctoral student (MScN) working as a research associate and Advanced Practice Nurse in long-term care, with over 5 years of experience conducting qualitative research – conducted all data collection. In Facility A, the researcher had established initial contact with nursing staff during an exploratory field phase (January 2024). No prior relationships existed with relatives or participants in Facility B. We disclosed professional background (research associate, APN) and dissertation context (study aim: understanding “being relative” in long-term care) during the consent process.

### Data Collection

Facility A served as the primary field site (January–April 2025), where we conducted 25 participant observations, 27 walking interviews, and 25 follow-up conversations. Facility B served as a verification site (May & June 2025), where we conducted 5 participant observations, 5 walking interviews, and 5 follow-up conversations to examine whether patterns from Facility A were context-specific and to assess thematic sufficiency.

We guided data collection using structured interviews and observation guides that were developed and pilot-tested during an exploratory phase (January 2024) with two nursing staff members and one relative at Facility A. This pilot phase verified the appropriateness of data-collection methods for maintaining naturalistic settings and established audio recording of post-encounter reflections as the primary documentation approach. They themselves required no modifications. Guides for walking interviews and follow-up conversations included open-ended questions that explored relational, spatial, and temporal dimensions while remaining flexible to allow participant-initiated topics. Observation guides used reflexive prompts focused on these exact dimensions. All guides included reflection sections for documenting themes, impressions and developing questions.

*Participant observations:* We conducted 30 observations totalling approximately 90 hr (each ~3 hr during afternoon visiting times) following Spradley’s (2013) three-phase approach. As participant-as-observer (Gold, 1958), the researcher focused on interactions between relatives and nursing staff, with residents sometimes present as part of the interactional context. To minimise intrusiveness, we made no audio recordings during observations; instead, the researcher took handwritten notes and, immediately afterwards, audio-recorded detailed reflections. We transcribed these recordings into structured fieldnotes that included initial interpretations. We later integrated fieldnotes with interview and conversation data to create comprehensive documents for each encounter.

*Follow-up conversations with relatives:* We conducted 30 conversations (30–60 min each) shortly after observed encounters, inviting relatives to reflect on what mattered during the encounter. Documentation followed the same process as observations: the researcher took brief notes during conversations, followed by audio-recorded reflections, which were transcribed into structured fieldnotes.

*Walking interviews with nursing staff:* We conducted 32 interviews (15–25 min each) as we moved through the physical environment, enabling context-bound reflection on practice and relationships. This approach was more consistent with the nursing staff’s everyday work patterns than interrupting their work for seated interviews in meeting rooms, thereby maintaining more naturalistic conditions. Documentation followed the same process, with all conversations and interviews conducted one-to-one.

### Data Analysis

**Figure 1.**
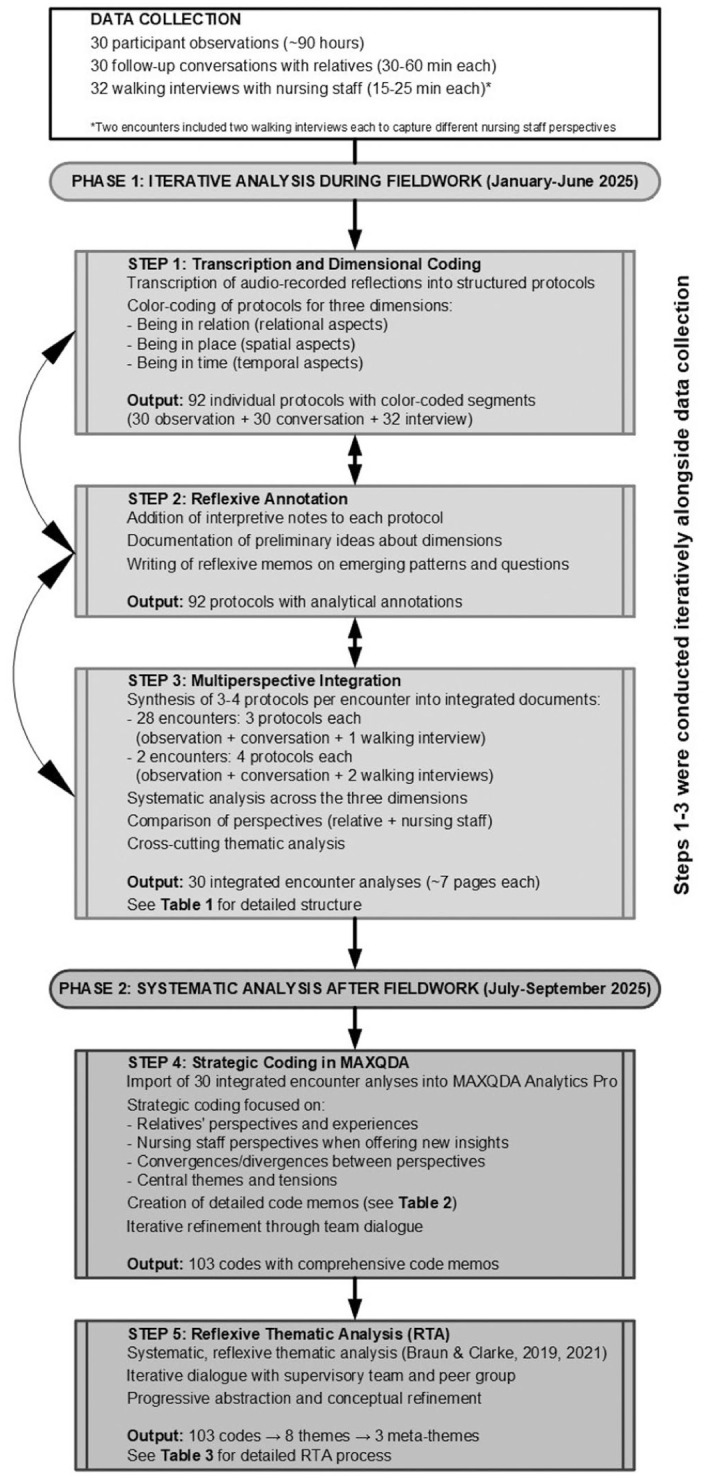
Analytical process overview. *Note*. The process was organised into two phases: iterative analysis during fieldwork (Steps 1–3) and systematic analysis after fieldwork was completed (Steps 4–5). Each step was built systematically on the previous one, enabling progressive refinement and conceptual abstraction from raw data to theoretical dimensions.

We transcribed all audio recordings verbatim and analysed the data using Reflexive Thematic Analysis (Braun & Clarke, 2019, 2021; Byrne, 2022) in an iterative process. As illustrated in Figure 1, the analysis moved from fieldwork-embedded steps to systematic post-fieldwork synthesis, prioritising “progressive abstraction” to elevate findings from descriptive accounts to conceptual dimensions.

*Phase 1: Iterative Analysis During Fieldwork* To capture the complexity of each encounter, we developed a novel analytical unit: the *Integrated Encounter Analysis* (IEA). Instead of analysing isolated transcripts, we synthesised the observation fieldnotes, the relative’s follow-up conversation, and the nursing staff’s walking interview into a single, multi-perspective document for each of the 30 encounters. This integration followed a standardised structure (Table 2) designed to systematically compare perspectives across the three dimensions of being (relation, place, time) while preserving the situational context. We colour-coded these 92 initial fieldnotes dimensionally before synthesising them into the 30 IEAs, which served as our primary data corpus. Crucially, the IEA structure was designed to make convergences and divergences between perspectives analytically productive rather than treating them as problems to be resolved. For each dimension of being (relation, place, time), we systematically documented where perspectives aligned, where they diverged, and where the observation revealed dynamics that neither participant had articulated. No analytical hierarchy was imposed between perspectives – the relative’s account did not override the staff member’s or vice versa. Rather, the guiding analytical question was consistently: what do these convergences, divergences, and blind spots reveal about the ontological, constitutive dimensions of “being relative” in the institutional context? Discrepancies were thus not coded as “contradictions” requiring resolution but as analytically generative moments that illuminated dimensions of the phenomenon – such as ambiguity, power asymmetry, or intersubjective gaps – that would remain invisible in mono-perspective data.

**Table 2.** Structure of Integrated Encounter Analyses.

Section	Content	Analytical purpose
1. Situational Description	- Narrative account of observed encounter - Key interactions and moments - Setting and temporal context	Provide contextual foundation for subsequent analysis
2. Analysis by Dimensions of Being	- Systematic analysis of each dimension (being in relation, being in place, being in time), with four analytical layers per dimension: - Observed interactional aspects - Relative’s perspective - Nursing staff perspective - Convergences and divergences	Systematic multi-perspective analysis
3. Cross-cutting Analysis	- Central themes - Recurring patterns - Particularities of this encounter - Tensions and ambiguities	Thematic condensation across dimensions
4. Integrative Reflection	- Integration of perspectives - Open questions for further inquiry	Interpretive synthesis and conceptual deepening
5. Methodological Reflection	- Notes on documentation quality - Process particularities - Researcher positioning	Reflexive quality assurance

*Note.* The standardised structure ensured systematic multi-perspective comparison across 30 encounters while preserving the situational particularity of each case. These documents served as the primary data corpus for subsequent coding.

*Phase 2: Systematic Analysis After Fieldwork* Using MAXQDA Analytics Pro 24, we conducted strategic coding of the 30 IEAs. Coding focused on identifying the *constitutive conditions* of “being relative” rather than cataloguing functional tasks. To ensure analytical rigour and theoretical grounding, we documented each of the 103 codes using a standardised code memo template (Table 3).

**Table 3.** Code Memo Template.

Component	Description	Example
Definition/Description	Conceptual articulation of the code and its meaning	“Being relative” as caring expertise: “Systematic evaluation of care quality based on biographical knowledge and continuous observation, expressing caring competence and claim to participate in care decisions”
Concrete Examples from Observations	Specific instances from integrated encounter analyses illustrate the code	“Wife evaluating nail care: “You did it very well again’; demonstrating husband”s well-groomed nails as evidence of good care quality”
Distinction from Similar Codes	Boundaries and differences from related or overlapping code	“Different from “epistemological competition” because it represents cooperative expertise rather than competing knowledge claims; different from “caring co-responsibility” because it focuses on knowledge-based evaluation rather than practical participation”
Context and Conditions	When, where, and under what circumstances the phenomenon occurs	“More pronounced among relatives with own care experience; intensifies when quality concerns arise; more frequent during critical health states”
Theoretical Connections	Links to the conceptual framework and relevant literature	“Experiential knowledge in nursing research; Mol’s co-production of care; lay-expert relationships in healthcare systems”

*Note.* This template was used to document each of the 103 codes. The components were designed to ensure analytical rigour and facilitate the shift from descriptive coding to theoretical grounding.

**Figure 2.**
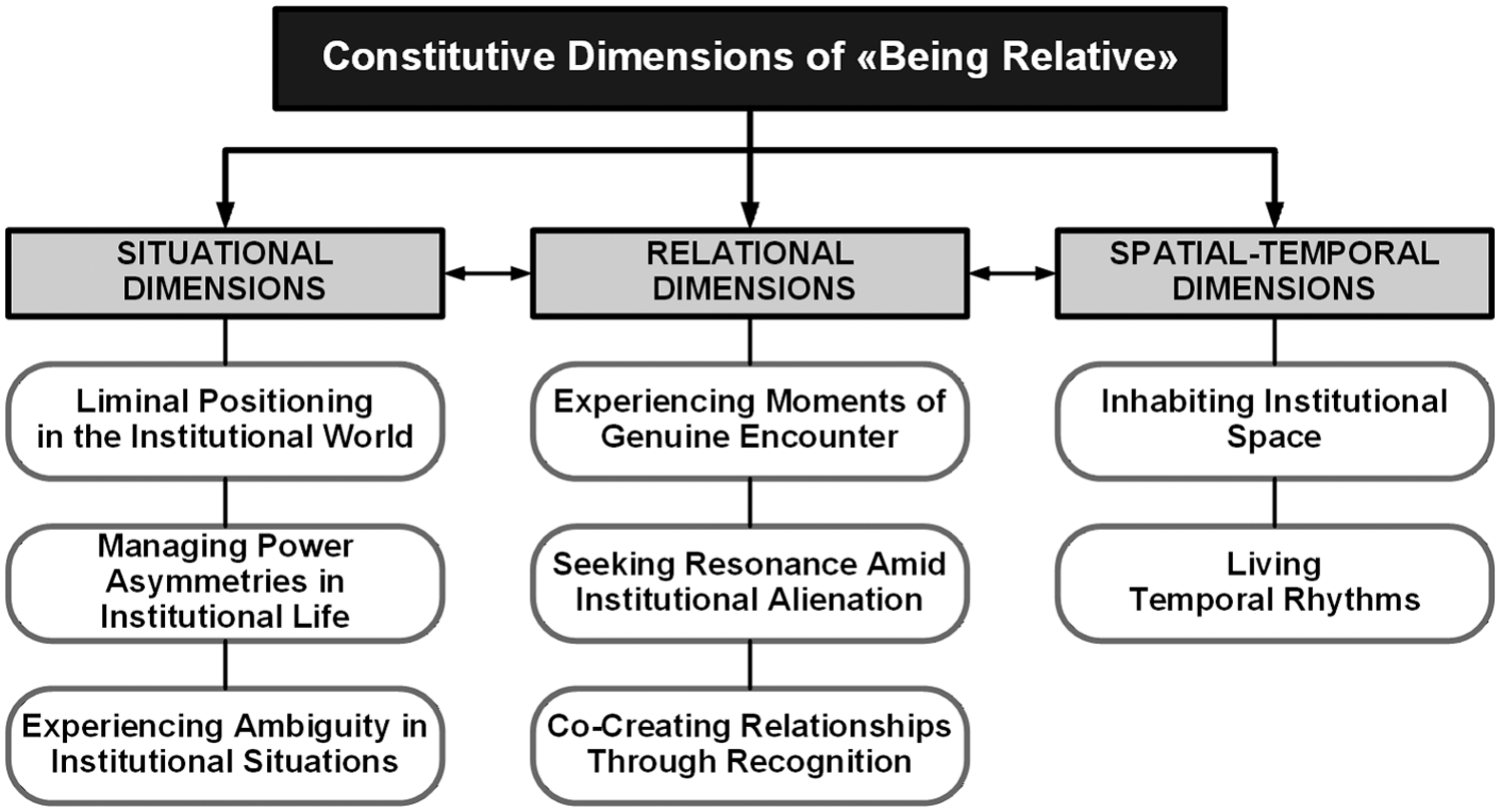
Constitutive dimensions of “being relative” in residential long-term care. *Note.* The framework presents eight sub-dimensions organised across three interconnected meta-dimensions (Situational, Relational, Spatial-Temporal). Bidirectional arrows indicate that dimensions do not operate in isolation but manifest simultaneously in any given encounter. Within each sub-dimension, relatives’ experiences moved dynamically between two poles – from institutional determination toward agency and appropriation.

Following Braun and Clarke’s six-phase approach (2019; Table 4), we clustered codes into candidate themes. Through recursive dialogue with the supervisory team and peer group, we refined these into eight themes and finally three meta-themes. This process involved an abductive theoretical dialogue in which we read the patterns we had constructed against established sociological and philosophical frameworks (i.e., Turner’s liminality, Goffman’s institutional analysis, and Buber’s encounter philosophy), enabling the progressive abstraction from concrete descriptions to the final ontological dimensions. The Results present these dimensions as empirical findings (Figure 2); the theoretical dialogue with Goffman, Turner, and Buber is developed in the Discussion.

**Table 4.** Reflexive Thematic Analysis Process.

Phase (Braun & Clarke)	Concrete activities in this study	Outputs/products
1. Familiarisation with data	- Repeated reading of 30 integrated encounter analyses (~210 pages) - Listening to audio recordings while reading transcripts - Writing reflexive memos on overall impressions - Noting recurring patterns, contradictions, and surprising elements	- Reflexive memos and initial notes integrated in fieldnotes - Preliminary ideas on patterns and tensions
2. Generating initial codes	- Systematic coding in MAXQDA guided by analytical questions (see methods text) - Strategic focus on relatives’ perspectives, convergences/divergences, and tensions - Creation of code memos for each code (see Table 2) - Iterative refinement through coding-recoding - Regular team meetings to discuss constructed codes and interpretations	- 103 codes with detailed code memos - Coded segments across 30 analyses - Theoretical memos on patterns
3. Generating initial themes	- Clustering of codes into provisional themes - Visual mapping of code relationships using MAXQDA tools and hand-drawn diagrams - Writing initial theme descriptions - Examining patterns across the three dimensions (relation, place, time) - Team discussions to test theme coherence	- Initial thematic map with 5 candidate themes - Visual maps of code relationships - Theme memos documenting rationale
4. Developing and reviewing themes	- Iterative refinement through multiple cycles - Development of meta-theme structure from 5 candidate themes - Progressive organisation into a hierarchical structure - Checking themes against coded data extracts - Extended team dialogue on theme boundaries and relationships - Presentation to doctoral peer group for critical feedback	- Final structure: - 3 meta-themes with - 8 sub-themes - Revised thematic map - Detailed theme descriptions - Documentation of decisions
5. Refining, defining and naming themes	- Writing detailed definitions for each theme - Identifying theoretical frameworks within themes at different levels of engagement (Turner, Goffman, Buber, Lefebvre, Budner, Rosa, Honneth) - Examining relationships between themes - Organising sub-themes within meta-themes based on conceptual relationships - Developing theme names that captured the essence - Creating a visual representation of the framework - Testing framework against data for fit	- 3 meta-themes: - Situational, - Relational, - Spatial-Temporal - 8 sub-themes organised within meta-themes - Conceptual framework diagram - Finalised theme definitions
6. Producing the report	- Selection of illustrative examples for each theme - Writing theme narratives that moved beyond description to interpretation - Weaving analytical narrative across themes - Ensuring coherence between background, analysis and findings - Iterative writing-revision cycles with team and co-authors	- Integrated results narrative - Conceptual framework (Figure 2) - Manuscript for publication

*Source*. Based on Braun and Clarke (2019, 2021).

*Note.* The process was iterative and recursive rather than linear, characterised by ongoing movement between phases as the analysis deepened through team dialogue.

### Trustworthiness

We ensured trustworthiness through triangulation of three data sources, prolonged engagement (6 months), iterative analysis, and comprehensive documentation of fieldnotes, code memos and analytical decisions. Thick description of settings, participants and contexts supports transferability (Benner, 1994), while explicitly documented pre-understandings strengthen confirmability (Geanellos, 1998a, 1998b).

Following established practices in qualitative nursing research for examining the researcher’s positioning (Benner, 1994; Geanellos, 1998a, 1998b), the researcher systematically documented his pre-understanding before and during fieldwork. This included documenting personal and professional experiences with relatives in long-term care, theoretical assumptions about person-centred care and “good” family involvement, and anticipated challenges such as role ambiguity and institutional constraints. We embedded reflexive practice directly within data collection and analysis procedures rather than maintaining it as a separate research diary. This approach ensured that reflexive thinking occurred in situ, closely tied to specific observations and analytical moments.

We continued data collection until we achieved thematic sufficiency (Braun & Clarke, 2019, 2021) meaning we had sufficiently rich data to understand the complexity and variation in experiences. Following Hennink et al.’s (2017) concept of “meaning saturation,” we assessed sufficiency by our ability to articulate coherent narratives for each theme and confirm through data from Facility B that patterns were not context-specific.

Crucially, we aligned our methodological conduct with person-centred research principles (Jacobs et al., 2017). Rather than treating these as abstract concepts, we operationalised them directly within the fieldwork to address relational complexities:

Connectedness: We established connectedness by reducing the distance between “researcher” and “participant.” This involved creating a relaxed atmosphere through humour, showing genuine interest in participants’ biographies beyond the immediate research topic, and occasionally assuming minor supportive tasks on the ward. This approach signalled that the researcher was not detached from the reality of care but was actively present within it.

Mindfulness and Dialogue: We practised mindfulness by continuously gauging the ethical boundaries of observation. For instance, the researcher voluntarily withdrew from intimate situations – such as a sensitive conversation between a nurse and a daughter regarding a resident’s approaching end-of-life – prioritising the dignity of the moment over data collection. Dialogue was fostered by creating a communicative space in which participants felt safe speaking freely.

Empowerment and Participation: To address power imbalances, we actively supported empowerment by validating participants as the primary experts of their experience. We explicitly encouraged relatives and staff to describe “what mattered” to them in their own words. We affirmed that their subjective view was the gold standard for this study, thereby shifting authority from the researcher to the participant.

Reflexivity: Reflexivity served as the integrative mechanism to monitor these dynamics. By systematically documenting his own positioning, the researcher critically examined how his professional background and presence influenced the field, ensuring that the principles of connectedness and empowerment were consistently upheld. This included reflecting on how his gender as a male researcher may have shaped interactional dynamics in a predominantly female field, both among nursing staff (9 women, 1 man) and relatives. While gender can function as a facilitating factor in establishing initial contact, the researcher’s everyday professional experience as a male nurse in long-term care – where both staff and residents are predominantly female – meant that navigating these dynamics was not unfamiliar but part of his habitual professional practice. Nevertheless, we remained attentive to the possibility that gender dynamics may have influenced which topics participants raised or avoided, particularly regarding themes of power and emotional ambiguity. Consistent with reflexive-constructivist epistemology (Braun & Clarke, 2019), we understood themes as researcher-generated interpretations grounded in sustained engagement with data and dialogue with the supervisory team.

### Ethics

The Ethikkommission Ostschweiz (EKOS) determined that the study did not fall under the Human Research Act because it does not involve research on diseases or bodily functions (Req-2024-01227; EKOS 24/170). The study adhered to the principles of the Declaration of Helsinki and the Swiss Human Research Act (HRA).

All participants provided written informed consent. We focused our observations on relatives’ and staff behaviours rather than residents’ private information. We paid particular attention to residents with cognitive impairment and were prepared to discontinue observations if residents showed distress, though this did not occur. We protected confidentiality through pseudonyms, generalised facility details, and secure data storage on encrypted devices.

## Results

Analysis of 30 Integrated Encounter Analyses (IEAs) revealed eight constitutive dimensions of “being relative” in residential long-term care, organised into three interconnected meta-dimensions: Situational (liminality, ambiguity, power), Relational (genuine encounters, resonance, co-creation), and Spatial-Temporal (space appropriation, temporal rhythms; Figure 2). These existential conditions manifested through empirically inseparable dimensions: in any given encounter, multiple dimensions operated simultaneously. The framework directs attention not to functional roles or participation patterns, but to the existential conditions under which “being relative” unfolds in institutional contexts. Across all eight dimensions, “being relative” manifested as a dynamic condition that unfolded between two poles. At one pole, relatives experienced institutional determination – constraint, powerlessness, functional reduction, and spatial and temporal subjection. At the other pole, they enacted agency – appropriation, self-determination, relational co-creation, and meaningful inhabitation of space and time. “Being relative” is this continuous movement between poles: the same relatives shifted positions from encounter to encounter, and often within moments, reflecting the processual, co-produced nature of institutional presence. The dimensions thus describe not person types but dynamic existential states.

Before presenting the analytical dimensions, we offer a composite narrative (Box 1) that illustrates how “being relative” manifests in everyday institutional life. While constructed from multiple observed encounters across facilities and anonymised for confidentiality, it conveys the lived texture of relatives’ institutional presence – the simultaneity, ambiguity, and relational complexity that the subsequent analytical dimensions seek to capture. The narrative makes visible what the dimensional framework formalises analytically: the eight dimensions do not operate in isolation but manifest together in any given encounter, with relatives moving across continua within moments – a processual, co-produced presence against which the dimensions described below should be read. We invite readers to return to this narrative after engaging with the dimensions, as the interplay between concrete experience and analytical abstraction is central to understanding “being relative.”

**Box 1.** “Being Relative” in Practice – A Composite Narrative.

The heavy entrance door closes behind Anna. The smell of disinfectant and coffee marks the boundary between outside and inside, between belonging and foreignness. Anna manoeuvres to her familiar place at the threshold – this place of conscious in-betweenness, where invisible rules determine where she may be and where not. She waits for twenty minutes, fitting into rhythms that are not hers. The signals are challenging to interpret: a brief nod whilst passing by, a “just a moment” without a time specification, glances meant for her but that do not reach her. Anna senses the hierarchy of urgencies in which she ranks far below. Waiting becomes a skill, an interpretation of a daily puzzle. Then a nursing staff member stops beside her, sits down with her and says: “You know, your mother often speaks of you. She is proud of you.” Anna feels something opening within her – being seen, being valued. As they both laugh about a peculiarity of Anna’s mother, a connection suddenly emerges. Two people who care about the same person understand each other for a moment without words. It lasts only seconds, but Anna carries it with her. After the conversation, Anna does something she would never have dared earlier. Instead of going immediately to her mother’s room, she walks to the lounge at the end of the corridor. She has claimed this space for months – not through asking or permission, but through persistent presence. Here she can be undisturbed with her mother, here she determines the time herself. “When I come, I have time,” she had once told the nursing staff member. Today, she stays longer than usual and feels no pressure. On the way back, she reencounters the nursing staff member in the corridor. A brief exchange – hasty, interrupted by an alarm. Anna nods understandingly. She has learnt that conversations can end abruptly, that institutional priorities have their own logic. However, she has also learnt to create moments and inhabit spaces. Some visits are characterised by waiting, others by intense encounters that last only minutes but change everything. What Anna experiences fundamentally characterises “being relative” in care facilities. “Being relative” proves to be a complex achievement between multiple dimensions of institutional existence: situationally manoeuvring between belonging and foreignness, relationally oscillating between functionality and genuine encounter, spatially-temporally oscillating between institutional determination and self-determined appropriation.

### Situational Dimensions of “Being Relative”

Across observed encounters, we identified three situational dimensions as fundamental conditions shaping relatives’ institutional presence: permanent in-betweenness and border-crossing (Liminal Positioning), daily confrontation with ambiguity and uncertainty (Experiencing Ambiguity), and structural disadvantage arising from institutional power asymmetries (Managing Power Asymmetries). Together, these dimensions constitute the situational foundation within which “being relative” manifests.

#### Liminal Positioning in the Institutional World

“Being relative” manifested fundamentally as threshold existence between institutional and private worlds. Across observations, relatives experienced permanent in-betweenness – a threshold existence that produced fragile belonging and latent uncertainty in the everyday encounter.

At one pole, relatives experienced institutional permanent liminality. One wife described how she “*sat quietly and expectantly in the hallway*” (IEA, 22 March 2025), consciously choosing not to appear demanding. Field observations repeatedly showed relatives in threshold positions: *waiting at doors, in transitional areas, between professional and familial spher*es (e.g., IEA, 8 February 2025). *Requests to leave rooms during care procedures* intensified this experience of exclusion alongside emotional connectedness (e.g., IEA, 14 February 2025).

At the other pole, relatives developed situational competencies. One wife explained “*that she deliberately always positioned herself at the doorway threshold*” (IEA, 23 March 2025) to initiate interactions without crossing boundaries. Relatives transformed threshold locations into encounter spaces – *entrance areas became places for conscious farewells* (e.g., IEA, 5 April 2025). Despite these competencies, the threshold position remained: relatives continuously oscillated between belonging and alienation.

#### Experiencing Ambiguity in Institutional Situations

“Being relative” manifested as ongoing interpretive work in situations where the same interactions simultaneously conveyed contradictory meanings. Across encounters, relatives confronted permanent ambiguity – signals of recognition and disregard, closeness and distance, trust and mistrust overlapped, creating continuous interpretive work and emotional ambiguity.

At one pole, relatives experienced ambiguity as threatening. One wife described her uncertainty “*whether she was being a burden or whether she was justified in seeking help*” (IEA, 22 February 2025). Field observations showed relatives in situations where the same interaction was experienced simultaneously as “*superficially friendly*” yet crossing “*a personal boundary*” (IEA, 6 April 2025). This ambiguity generated continuous interpretative work.

At the other pole, relatives developed the ability to accept contradictory signals. One daughter described the relationship with a nursing staff member as “*fundamentally positive and appreciative*”, but simultaneously noted that “*something always resonated*” (IEA, 15 March 2025) – scepticism or control. The balance between professional distance and personal closeness, particularly visible in *divergent evaluations of informal address* (IEA, 1 February 2025), was interpreted as a usual accompaniment of institutional relationships. Relatives found security in accepting this openness of interpretations.

#### Managing Power Asymmetries in Institutional Life

“Being relative” manifested as continuous manoeuvring through structural power asymmetries. Across encounters, relatives’ presence was characterised by asymmetric power relations in which institutional routines, temporal regimes, and hierarchical structures shaped what was possible – and what required strategic manoeuvring.

At one pole, relatives experienced structural powerlessness. One wife described that she had “*asked about the shirt 3 weeks ago*” (IEA, 14 February 2025) but had not received a response. Field observations showed relatives waiting while nursing staff passed by without noticing them – one nursing staff member “*only noticed by chance that the wife was sitting on the bench in the hallway*” (IEA, 22 March 2025). In another observation, a daughter “*behaved quietly and waited*” (IEA, 26 April 2025) while nursing staff attended to other residents. Institutional prioritisation fundamentally limited their ability to act.

At the other pole, relatives developed calculated strategies. One son described the timing of his intervention as “*very appropriate*”, demonstrating “*strategic time management in communication*” (IEA, 15 March 2025). One wife, after lengthy waiting, “*went to check herself*” whether her husband was in bed (IEA, 14 February 2025). These strategic interactions enabled them to unlock spaces for action despite structural constraints. The power asymmetry was intermittently subverted, without being entirely abolished.

### Relational Dimensions of “Being Relative”

Across observed encounters, we identified three relational dimensions as fundamental conditions shaping relatives’ interpersonal experiences: fleeting moments of authentic encounter beyond functional interactions (Experiencing Genuine Encounters), the search for genuine resonance despite institutional alienation (Seeking Resonance), and co-creative shaping of recognition moments (Co-Creating Relationships). Together, these dimensions constitute the interpersonal foundation within which “being relative” manifests as continuous oscillation between everyday interactions and transformative encounters.

#### Experiencing Moments of Genuine Encounters

“Being relative” manifested in the relational quality of everyday encounters with nursing staff. Across observations, the texture of these encounters varied considerably in their depth and immediacy.

At one pole, interactions remained functional and routinised. Field observations showed a *friendly greeting with a smile without physical contact* (IEA, 1 February 2025) – a positive but professionally distanced relationship. One observation documented a “routine encounter versus meaningful interaction” (IEA, 8 March 2025), in which relatives experienced encounters as *functionally efficient but relationally distant*.

At the other pole, moments of immediate presence appeared. Field observations showed a “*brief touch of the hand*” during the farewell (IEA, 1 March 2025) – physical touch as a sign of relationship deepening. One wife described feeling “*seen and valued*” by the nursing staff member (IEA, 14 February 2025). Field observations showed “*immediate positioning at eye level with eye contact*” (IEA, 22 March 2025) – embodied presence transcending functional interaction. One relative valued the “*spontaneous hallway conversation*” (IEA, 17 May 2025) as particularly meaningful. These moments manifested through humorous connection, embodied resonance and empathic being-understood. Small gestures of nonverbal communication – a touch, a glance, shared gratitude – briefly transcended institutional positions. The fleetingness of these genuine encounters made them existentially significant.

#### Seeking Resonance Amid Institutional Alienation

“Being relative” manifested as a search for relational resonance in institutional relationships. Across encounters, relatives’ relational experiences moved along two axes: horizontal (social relationships with nursing staff) and vertical (self-identity as relatives in the institutional context).

At one pole, relatives experienced a “relationship of relationlessness”. One wife described her uncertainty about whether “*the nursing staff member had fully understood her and her concern*” (IEA, 1 February 2025). Another wife expressed scepticism “*whether the nursing staff were consistently using the ointment she used*” (IEA, 1 February 2025). Field observations showed *inner distancing versus outer attentiveness* – institutional communication fell silent despite many words (e.g., IEA, 8 March 2025).

At the other pole, moments of mutual transformation occurred. One wife particularly valued how “*the nursing staff member actively included her husband in the conversation with naturalness, fun and humour*” (IEA, 5 April 2025), and both sides were affected. Field observations showed how the nursing staff member’s *understanding and relaxed attitude* (e.g., IEA, 27 April 2025) enabled flexible responses to different situations. In these moments of *shared understanding* (e.g., IEA, 1 March 2025), the institutional choreography dissolved. These moments were fleeting but existentially significant. Their unavailability made them precious.

#### Co-Creating Relationships Through Recognition

“Being relative” manifested as a mutual, co-creative relationship formation mediated through shared concern for the care-dependent relative. Across encounters, relatives’ relational experiences took form through co-creative shaping of relationships with nursing staff.

At one pole, relatives remained trapped in their function. One wife described her efforts as “*very protective and sheltering towards her husband*” (IEA, 1 February 2025), which nursing staff perceived as “*sometimes quite demanding*” (IEA, 1 February 2025) – her expertise was not recognised. Field observations showed relatives’ *claim to co-create rather than a passive spectator role* (e.g., IEA, 2 February 2025), yet their *wishes for active involvement* were not fulfilled. The importance of “*being perceived as a person and not only as a function bearer*” (IEA, 2 March 2025) highlighted how relatives felt reduced to their role – no genuine relationship formed.

At the other pole, co-creatively shaped relationships grew. One wife experienced “*a partnership relationship with the nursing staff member rather than a pure service relationship*” (IEA, 1 March 2025). Field observations documented *mutual appreciation of respective roles and contributions* (e.g., IEA, 1 March 2025). Another wife valued the “*personal connection through shared history*” (IEA, 5 April 2025) – the nursing staff member had previously cared for her own mother. Observations showed *participative care as a shared field of action* (IEA, 17 May 2025), where relatives were actively involved. This mutual valuing developed a shared relational practice with its own rituals.

### Spatial-Temporal Dimensions of “Being Relative”

Across observed encounters, we identified two spatial-temporal dimensions as fundamental conditions shaping relatives’ institutional presence: spatial positioning between institutional determination and self-determined space appropriation (Inhabiting Institutional Space), and temporal synchronisation between prescribed rhythms and self-determined time management (Living Temporal Rhythms). Together, these dimensions constitute the structural infrastructure within which “being relative” manifests.

#### Inhabiting Institutional Space

“Being relative” manifested as an ongoing spatial practice within institutionally conceived space. Across observations, relatives positioned themselves, navigated institutional spaces, and at times transformed them – producing their own inhabited, lived space within these surroundings.

At one pole, one wife “*deliberately positioned herself at the door opposite the table where her husband sat, at a distance of approximately three metres*” (IEA, 22 February 2025). When care activities were interrupted, “*immediate apology and withdrawal*” occurred (IEA, 8 March 2025). One wife observed activities in the staff room, and she gained the “*impression that nursing staff were busy*” (IEA, 22 February 2025). Another observation, precise positioning “*six metres from the room door*” in public space (IEA, 9 March 2025), demonstrated spatial accommodation.

At the other pole, “*purposeful seeking of the dining room by the wife showed good orientation and familiarity with the premises*” (IEA, 1 February 2025). One daughter “*moved independently between various rooms (dining room, mother’s room, corridor)*” (IEA, 22 February 2025) and used “*the corridor opportunistically as a spontaneous communication space*” (IEA, 22 February 2025). Relatives transformed institutional places – “*transformation of institutional spaces through familial appropriation (entrance area as place of farewell)*” became a personal space of meaning (IEA, 23 March 2025).

#### Living Temporal Rhythms

“Being relative” manifested as ongoing temporal practice within institutional time structures. Across observations, relatives’ temporal experiences were shaped at the intersection of institutional rhythms and their own.

At one pole, one wife patiently fitted into institutional time structures and accepted “*to sit down quietly and wait until her husband was ready*” (IEA, 22 March 2025). Waiting times were experienced as “*a natural part of the temporal arrangement*” (IEA, 8 March 2025) without frustration or resistance. One daughter demonstrated pronounced adaptability and “*accepts flexibly and understandingly that conversations sometimes must be ended abruptly*” (IEA, 26 April 2025) when care priorities demanded this.

At the other pole, one wife emphasised her own temporal sovereignty: “*When she comes to visit, she has time*” (IEA, 22 March 2025) – a conscious decision for “*flexible, situative time shaping without strict planning*” (IEA, 9 February 2025). Another wife experienced unexpected moments as existentially significant – “*strong emotional charging*” when her husband walked in the afternoon, although this “*normally only happens in the morning*” (IEA, 9 March 2025). “*Brief, but intense interaction between all participants*” (IEA, 16 March 2025) was transformed through mutual presence into condensed moments.

## Discussion

### Summary of Key Findings

This study conceptualises “being relative” in residential long-term care as an existential condition characterised by permanent threshold existence, continuous interpretive work, and negotiated agency within institutional constraints. Through analysis of 30 Integrated Encounter Analyses (IEAs), we identified constitutive dimensions, organised into three interconnected meta-dimensions: Situational, Relational, and Spatial-Temporal. Critically, these dimensions manifested not as static attributes but as dynamic continua along which the same relatives moved situationally – from liminal to liminoid positioning, from alienation to resonance, from institutional subjection to strategic agency. “Being relative,” the findings reveal, is dynamic: relatives’ identities and experiences are formed through continuous oscillation across multiple dimensions operating simultaneously in any given encounter.

This conceptualisation challenges prevailing frameworks that position relatives merely as visitors, participants, or partners – functional categories that obscure the existential dimension of institutional presence. Rather than asking how relatives participate or what roles they perform, this framework directs attention to the conditions under which “being relative” unfolds: the threshold spaces they inhabit, the ambiguity they manage, the power asymmetries they manoeuvre through, and the resonance they seek. These findings are discussed in relation to three complementary theoretical perspectives – Goffman’s sociology of institutions, Turner’s theory of liminality, and Buber’s philosophy of encounter – in the Theoretical Contributions section below.

### Theoretical Contributions

Drawing together the empirical findings presented above, our analysis moves beyond functional descriptions of family involvement to conceptualise “being relative” as a distinct ontological condition. Three complementary theoretical perspectives illuminate different facets of this condition: Goffman’s (1961, 1969) sociology of institutions reveals the conditions that produce it, Turner’s (1969, 1982) theory of liminality captures how it is existentially experienced, and Buber’s (1923) philosophy of encounter illuminates what is at stake in the dyadic relationships through which it unfolds.

*The institutional conditions: Goffman.* Goffman’s analysis of total institutions provides the structural explanation for the conditions under which “being relative” unfolds. Although residential long-term care facilities are not total institutions in Goffman’s strict sense, our findings demonstrate that institutional effects radiate beyond formal boundaries. Relatives, who remain formally outside the institution’s jurisdiction, nevertheless experienced its power asymmetries, temporal regimes, and hierarchical structures as fundamentally constraining. The institution controlled space (where relatives could be, when they had to leave), time (waiting, interruption, scheduling), and interaction (who spoke first, what could be addressed, how concerns were received). This radiating institutional power created the conditions under which all eight dimensions identified in this study – situational, relational, and spatial-temporal – manifested and operated. Goffman thus functions as the contextualising framework that explains *why* relatives found themselves in the existential condition described by the findings.

*The existential experience: Turner.* Turner’s concepts of liminality and liminoidality capture *how* relatives experienced their institutionally conditioned position. Originally theorised as a temporary ritual threshold through which initiands pass toward reintegration, liminality in our findings manifested as a permanent existential condition: relatives did not transition through the threshold but became permanent dwellers within it – “constant commuters between worlds” who never fully arrived on either side. This permanent in-betweenness pervaded not only the situational dimensions (threshold positioning, ambiguity, powerlessness) but also the spatial-temporal dimensions: relatives inhabited institutional spaces without ever fully belonging to them and experienced institutional time as a rhythm imposed from without. The liminoid pole – Turner’s concept for voluntary, creative, and agentive threshold experiences – captured the moments when relatives developed competencies, appropriated spaces, and claimed temporal sovereignty within institutional constraints. The continuous oscillation between liminal and liminoid poles that our findings documented across all dimensions suggests that “being relative” constitutes a form of permanent liminality in which the promise of reintegration – central to Turner’s original ritual model – is structurally withheld.

*The relational encounter: Buber.* While Goffman and Turner illuminate the structural conditions and existential experience of “being relative,” neither theorises the quality of what unfolds within the dyadic encounter itself. Buber’s distinction between I-Thou and I-It relations addresses precisely this dimension. Our findings revealed that, across the relational meta-dimension, the fundamental question was whether relatives and nursing staff encountered one another as persons or as functions. At one pole, interactions remained instrumental – professionally correct but relationally empty, reducing the relative to a role bearer and the nursing staff member to a service provider. At the other pole, moments of genuine encounter manifested: brief, often nonverbal moments of mutual presence in which institutional positions dissolved and persons met as persons. These encounters were scarce, fleeting, and unpredictable – yet they served as “anchor points of personhood” sustaining relatives’ existential endurance within the institution. The scarcity of genuine encounter was itself a product of the institutional conditions Goffman describes: it was the functional organisation of care that made I-Thou moments exceptional rather than ordinary. Buber thus reveals what is existentially at stake in the relational dimensions – and why the quality of the dyadic encounter cannot be reduced to structural or procedural explanations.

Taken together, these three perspectives demonstrate that “being relative” constitutes a distinct ontological condition – not a functional variant of “visitor” or “partner,” but a mode of institutional presence characterised by permanent dwelling in the in-between. The institutional context (Goffman) produces the conditions, the threshold experience (Turner) characterises how these conditions are lived, and the dyadic encounter (Buber) reveals what remains existentially possible – and precarious – within them. This triadic theoretical architecture mirrors the phenomenon’s own structure: “being relative” is simultaneously conditioned, experienced, and relationally enacted.

### Implications for Practice

Based on the findings and their theoretical integration, we identify concrete implications for how institutions can recognise and support relatives’ presence in residential long-term care. The three theoretical perspectives structuring the discussion – institutional conditions (Goffman), threshold existence (Turner), and dyadic encounter (Buber) – each generate distinct practical implications.

#### Making Institutional Conditions Visible and Negotiable

The finding that institutional effects radiate beyond formal boundaries to structure relatives’ experience suggests that institutions must first acknowledge the power asymmetries they produce. Institutional communication must acknowledge these asymmetries explicitly. This includes establishing explicit guidelines about presence during care activities to reduce the interpretive burden on relatives. This does not mean eliminating prioritisation, but making power relations visible and negotiable. Simple practices – greeting relatives by name, explicitly acknowledging waiting times, and proactively sharing temporal structures – can shift the experience from subjection toward agency.

Since some ambiguity is inherent to institutional life (e.g., fluctuating resident health, shifting routines), practice should acknowledge this reality explicitly rather than presenting contradictions as failures. Practice development programmes should incorporate “ambiguity management” to help nursing staff understand how their mixed signals – such as professional warmth coupled with abrupt temporal constraints – create ongoing interpretive work for relatives. Where contradictory signals are unavoidable (because care is both personal and professional), making these tensions explicit helps relatives develop contextual interpretation skills.

#### Recognising and Supporting Threshold Existence

The finding that relatives occupy permanent threshold positions challenges institutional frameworks that force binary categorisations. Traditional policies often position relatives as either “involved” (participating in care) or “uninvolved” (passive visitors), overlooking the legitimate presence of those who dwell in the in-between. Staff training should explicitly address threshold positioning, helping nursing staff understand that relatives who “just sit” in hallways are not passive observers but are actively enacting “being relative.” Institutions should reconceptualise spaces to accommodate threshold existence – creating areas that are neither clinical waiting rooms nor purely private zones, but recognised “zones of relative presence” where hovering and observing are validated as legitimate forms of “being there.” Practice should encourage opportunities for spatial appropriation, such as permitting the transformation of “public” spaces (corridors, balconies) into temporary personal territories for farewells or conversations.

Temporal implications challenge rigid adherence to schedules. Solutions might include flexible “windows of opportunity” rather than fixed visiting hours. Fundamentally, honouring threshold existence means viewing relatives’ daily movements and waiting times not as incidental behaviours, but as meaningful practices through which they negotiate their belonging within the institution.

#### Creating Conditions for a Genuine Encounter

The finding that genuine encounter is scarce yet existentially significant suggests that institutions cannot manufacture authentic connection, but can create the conditions that make it possible. Aligned with person-centred care frameworks (McCormack & McCance, 2021), structures must support relationship continuity – such as consistent staff-family pairings – to foster the shared histories necessary for genuine encounter. Organisations should explicitly acknowledge relatives’ expertise: small gestures – noting relatives’ observations in documentation or including their knowledge in handovers – serve as anchor points that validate their specific personhood and open possibilities for co-creative relationships. Practice must value fleeting encounters. Staff should be encouraged to recognise that a brief, authentic moment of eye-level contact or shared humour is not “inefficient time,” but an existentially significant anchor for the relative’s identity. The scarcity of these moments – a direct consequence of institutional conditions – makes each one count.

### Methodological Reflections

The methodological innovation of this study lay in the rigorous application of Integrated Encounter Analyses (IEAs). By synthesising participant observation, walking interviews with staff, and follow-up conversations with relatives into single analytical units, we moved beyond the limitations of mono-perspective data to capture the ontological complexity of “being relative” in three ways.

First, the IEAs revealed the “invisible” gap between intention and experience. While interviews often produce harmonious narratives (“good relationship”), our triadic data revealed discrepancies between professional intent and relatives’ lived experience. For instance, an interaction described by staff as “humorous” was often experienced by relatives as “dismissive” due to timing. The IEA format forced these discrepancies to surface, allowing us to identify “managing ambiguity” not just as a concept, but as an observable practice performed in the discrepancy between sent and received signals. To illustrate: in one encounter, a staff member described the interaction as relaxed and harmonious, while the relative expressed uncertainty about whether she was being a burden. The observation fieldnotes, in turn, documented spatial positioning and timing cues that neither participant had mentioned. Rather than privileging one account, the IEA structure enabled us to recognise this very discrepancy as constitutive of “being relative” – the persistent gap between observable harmony and experienced uncertainty manifested as a defining feature of institutional ambiguity. The code “intersubjective discrepancy,” which appeared across the majority of IEAs, captures precisely this analytical move: divergent perspectives were not resolved into a single “true” account but were read as evidence of the ontological conditions under which relatives exist in institutional settings.

Second, the method materialised the spatial-temporal dimension. While interviews often detach experiences from context, the IEAs anchored every statement in its physical setting. Findings like “liminal positioning” (hovering in doorways) became visible only because the Walking Interviews and Observations were spatially mapped. Similarly, capturing staff reflections “in movement” proved superior to seated interviews, mirroring the restless temporal rhythm that shapes their interactions.

Finally, the IEA structure facilitated progressive abstraction. Organising data immediately into dimensions of Being (Relation, Place, Time) prevented the analysis from remaining at the level of descriptive storytelling and forced an immediate theoretical filtering. Furthermore, this structure supported reflexivity. As an APN researcher embedded in the field, the IEA served as a corrective mechanism, documenting how professional pre-understandings shaped observations – revealing, for example, that what looked like “passive waiting” to the clinical eye was actually an active form of “tactful presence.”

A further methodological reflection concerns the scope of the dyadic focus within the triadic reality of institutional care. We recognise that “being relative” is not constituted solely within the relative-staff dyad: the resident-relative and resident-staff dyads equally shape the existential conditions under which relatives exist in institutional settings. The findings of this study, therefore, represent an independent but deliberately partial contribution – illuminating one constitutive relational axis in depth while acknowledging that a fuller understanding requires investigation of the remaining dyadic constellations. At the same time, the depth of engagement enabled by this focused design – 30 systematically documented encounters analysed through Integrated Encounter Analyses – produced findings that stand on their own as a substantive contribution to understanding “being relative” in institutional long-term care. Future research within and beyond the BE-REAL programme will need to investigate the remaining dyads to complete the multiperspective picture.

### Limitations

Several limitations frame the boundaries of these findings, pointing toward directions for further inquiry. First, the study’s Swiss residential long-term care context shapes the transferability of findings. Unlike systems in which family involvement in institutional care is legally mandated (e.g., Germany’s Heimbeiräte) or regulated through care-planning requirements (e.g., the UK), Switzerland has no federal or cantonal legislation prescribing routine family involvement in nursing homes. The federal Erwachsenenschutzrecht (Art. 382–387 ZGB) obliges institutions to foster external relationships “as far as possible” without prescribing how they should do so. Relatives’ institutional position is thus structurally undefined – negotiated locally rather than legally anchored. This indeterminacy may amplify the liminal positioning, ambiguity, and power asymmetries identified in this study. In contexts where family presence is more culturally prescribed (e.g., Southern European settings) or more legally defined, these dimensions may manifest differently. Additionally, Switzerland’s cantonal decentralisation produces considerable institutional variation (Lussi et al., 2024), which means the two facilities represent distinct regional contexts within a heterogeneous system.

Second, methodological choices created boundaries around *which* relatives’ experiences were captured. The pragmatic sampling approach recruited relatives who visited regularly and were willing to participate in extended encounters – excluding those who visited rarely, those who chose not to engage with the research, or those whose language or cognitive capacities limited their participation in verbal reflection. Consequently, the findings may underrepresent the experiences of relatives who enact presence silently or those who express “being relative” through absence or withdrawal. Furthermore, the temporal design captured encounters as snapshots rather than trajectories. While this enabled depth within single encounters, the long-term evolution of relationships had to be reconstructed narratively rather than observed longitudinally.

Finally, as discussed in the Methodological Reflections, residents’ direct perspectives were not captured through interviews. While observation documented residents’ presence and agency within encounters, future research should include residents’ subjective accounts to complement the dyadic findings presented here.

### Future Research Directions

These findings and their boundaries point toward several productive directions for further inquiry. First, longitudinal research could illuminate how “being relative” evolves – from initial institutionalisation through progressive decline to end-of-life. Such studies would capture the trajectories that our snapshot design could only reconstruct narratively, revealing how relatives’ positioning shifts across transitions and how relationships develop over years.

Second, comparative research across cultural and institutional contexts could test the transferability of these dimensions. Studies in settings with different family involvement norms, alternative care models (e.g., small-scale living arrangements), or diverse care settings (e.g., home care) would reveal which dimensions are universal to institutional presence and which are context-specific.

Third, intervention research – ideally using participatory designs – could test the practice implications developed here. Studies might co-design and evaluate training programmes focused on “ambiguity management,” organisational initiatives to create recognised “threshold spaces,” or policy changes implementing flexible “windows of opportunity.” Such research would move from *understanding* “being relative” to actively *supporting* it within institutional constraints.

Finally, methodological innovation could address this study’s limitations. Research including residents’ direct perspectives – through adapted communication methods for those with cognitive impairments – would complete the multiperspective picture. Additionally, exploring how digital technologies mediate “being relative” (e.g., hybrid presence through video calls) represents an emerging spatial-temporal dimension requiring investigation.

## Conclusion

This study conceptualises “being relative” in residential long-term care as an existential condition constituted through dynamic movement across eight interconnected dimensions. Rather than positioning relatives merely as visitors requiring integration or partners requiring activation, the findings direct attention to the existential reality of their presence: the threshold spaces they inhabit, the ambiguity they manage, the power asymmetries they manoeuvre through, the resonance they seek, the recognition they co-create, the encounters they experience, and the spatial-temporal agency they negotiate. These dimensions manifested not as static attributes but as continua along which relatives move situationally – “being relative” is this dynamic movement, not a fixed position.

The theoretical contribution lies in demonstrating how the institutional context fundamentally shapes the existential condition of “being relative”: institutional power radiates beyond formal boundaries to structure relatives’ experience (Goffman), liminality becomes a permanent dwelling rather than a transitional passage (Turner), and genuine encounter becomes a scarce yet existentially decisive possibility within functionally organised care (Buber). This triadic theoretical architecture suggests that “being relative” requires its own theoretical articulation – neither visitor nor insider, neither fully determined nor entirely free, but dwelling permanently in the in-between.

The practical significance extends person-centred care frameworks to recognise that relatives themselves require care that honours this existential condition. This means creating spaces for threshold existence rather than forcing categorisation, supporting ambiguity tolerance as a relational competency, and designing conditions for resonance rather than mandating connection. Understanding “being relative” as an existential condition opens possibilities for care that honours not only residents but also those who remain, paradoxically and permanently, both connected and separate, both inside and outside, both present and not-quite-here – like Anna at the threshold where being relative unfolds.
